# Deciphering the SOX4/MAPK1 regulatory axis: a phosphoproteomic insight into IQGAP1 phosphorylation and pancreatic Cancer progression

**DOI:** 10.1186/s12967-024-05377-3

**Published:** 2024-06-28

**Authors:** Chao Song, Ganggang Wang, Mengmeng Liu, Siyang Han, Meiyuan Dong, Maozhen Peng, Wenquan Wang, Yicun Wang, Yaolin Xu, Liang Liu

**Affiliations:** 1 Department of Pancreatic Surgery, Affiliated Zhongshan Hospital, Fudan University, No.180 Fenglin Road, Xuhui District, Shanghai, PR China; 2 Department of General Surgery, Qingpu Branch, Affiliated Zhongshan Hospital of Fudan University, Qingpu Branch, No. 1158 Park Road East, Qingpu District, Shanghai, PR China; 3 Shanghai Institute of Infectious Disease and Biosecurity, Fudan University, Shanghai, PR China; 4 Department of Hepatobiliary Surgery, Pudong Hospital, Fudan University, Shanghai, China; 5 Department of Gastroenterology, Qingpu Branch, Affiliated Zhongshan Hospital of Fudan University, Shanghai, PR China; 6 Department of Endocrinology, Shanghai Pudong Hospital, Fudan University, Shanghai, PR China

**Keywords:** Pancreatic cancer, Phosphoproteomics, Tumor typing, Tumor growth and metastasis, SOX4, MAPK1 kinase, IQGAP1 phosphorylation, KSEA analysis

## Abstract

**Objective:**

This study aims to elucidate the functional role of IQGAP1 phosphorylation modification mediated by the SOX4/MAPK1 regulatory axis in developing pancreatic cancer through phosphoproteomics analysis.

**Methods:**

Proteomics and phosphoproteomics data of pancreatic cancer were obtained from the Clinical Proteomic Tumor Analysis Consortium (CPTAC) database. Differential analysis, kinase-substrate enrichment analysis (KSEA), and independent prognosis analysis were performed on these datasets. Subtype analysis of pancreatic cancer patients was conducted based on the expression of prognostic-related proteins, and the prognosis of different subtypes was evaluated through prognosis analysis. Differential analysis of proteins in different subtypes was performed to identify differential proteins in the high-risk subtype. Clinical correlation analysis was conducted based on the expression of prognostic-related proteins, pancreatic cancer typing results, and clinical characteristics in the pancreatic cancer proteomics dataset. Functional pathway enrichment analysis was performed using GSEA/GO/KEGG, and most module proteins correlated with pancreatic cancer were selected using WGCNA analysis. In cell experiments, pancreatic cancer cells were grouped, and the expression levels of SOX4, MAPK1, and the phosphorylation level of IQGAP1 were detected by RT-qPCR and Western blot experiments. The effect of SOX4 on MAPK1 promoter transcriptional activity was assessed using a dual-luciferase assay, and the enrichment of SOX4 on the MAPK1 promoter was examined using a ChIP assay. The proliferation, migration, and invasion functions of grouped pancreatic cancer cells were assessed using CCK-8, colony formation, and Transwell assays. In animal experiments, the impact of SOX4 on tumor growth and metastasis through the regulation of MAPK1-IQGAP1 phosphorylation modification was studied by constructing subcutaneous and orthotopic pancreatic cancer xenograft models, as well as a liver metastasis model in nude mice.

**Results:**

Phosphoproteomics and proteomics data analysis revealed that the kinase MAPK1 may play an important role in pancreatic cancer progression by promoting IQGAP1 phosphorylation modification. Proteomics analysis classified pancreatic cancer patients into two subtypes, C1 and C2, where the high-risk C2 subtype was associated with poor prognosis, malignant tumor typing, and enriched tumor-related pathways. SOX4 may promote the occurrence of the high-risk C2 subtype of pancreatic cancer by regulating MAPK1-IQGAP1 phosphorylation modification. In vitro cell experiments confirmed that SOX4 promoted IQGAP1 phosphorylation modification by activating MAPK1 transcription while silencing SOX4 inhibited the proliferation, migration, and invasion of pancreatic cancer cells by reducing the phosphorylation level of MAPK1-IQGAP1. In vivo, animal experiments further confirmed that silencing SOX4 suppressed the growth and metastasis of pancreatic cancer by reducing the phosphorylation level of MAPK1-IQGAP1.

**Conclusion:**

The findings of this study suggest that SOX4 promotes the phosphorylation modification of IQGAP1 by activating MAPK1 transcription, thereby facilitating the growth and metastasis of pancreatic cancer.

**Supplementary Information:**

The online version contains supplementary material available at 10.1186/s12967-024-05377-3.

## Introduction

Pancreatic cancer is a prominent contributor to global cancer mortality, with a persistently low five-year survival rate [1]. The pathogenesis of pancreatic cancer is intricate and encompasses various genetic and environmental factors [2]. The continuous advancement of medical research has led to the recognition that protein phosphorylation modification plays a crucial role in the occurrence, development, and treatment of pancreatic cancer [3]. Phosphorylation is the predominant post-translational modification of proteins and contributes to various biological processes, including signal transduction, cell cycle regulation, and metabolic control [4–6].

Phosphoproteomics has emerged as a prominent field of research in recent years, offering a novel approach to elucidate phosphorylation events and their affiliated signaling pathways in cancer [7–9]. Phosphoproteomics uncovers the functions of diverse kinases and substrates in advancing tumors by examining protein phosphorylation sites [10]. SOX4, MAPK1, and IQGAP1 are all associated with the occurrence and progression of pancreatic cancer [11, 12].

SOX4 is a transcription factor involved in multiple biological processes, including cell proliferation, differentiation, and migration [13]. MAPK1, a kinase, is pivotal in numerous signaling pathways [14]. IQGAP1 is a versatile protein involved in cell cytoskeleton remodeling, cell migration, and intercellular signaling [15]. Although the individual functions of these proteins have been extensively studied, the interactions and synergistic effects in pancreatic cancer remain unclear.

This study aimed to investigate the role of IQGAP1 phosphorylation modification, which is mediated by the SOX4/MAPK1 regulatory axis, in the development of pancreatic cancer using phosphoproteomic analysis. We aim to offer novel strategies and a theoretical foundation for early diagnosis, molecular subtyping, and targeted pancreatic cancer therapy. This study will be achieved through a comprehensive analysis of tissues collected from patients with pancreatic cancer. This research is highly important for enhancing our understanding of the pathogenesis of pancreatic cancer and could facilitate the development of more precise and effective treatment methods for patients.

## Materials and methods

### Protein sequencing of pancreatic cancer tissue samples

Invitrogen’s protein extraction buffer (78,501) is utilized for extracting proteins from tissue samples, and the BCA method is employed to determine protein concentration. The protein samples were subjected to gel electrophoresis and digested with Invitrogen’s trypsin (15,400,054) under acidic conditions to break down the proteins into peptides. Following this, a mass spectrometer is utilized to analyze the sample. Pre-processing steps, including solid-phase extraction (SPE), are conducted to eliminate impurities. Following this, we utilized the liquid chromatography-mass spectrometry (LC-MS) instrument manufactured by Thermo Fisher to analyze proteins. Lastly, we examine the data obtained from mass spectrometry analysis of the protein, which comprises mass spectrum analysis, protein identification, quantitative analysis, and other relevant factors [16].

### Data downloading and analysis

The proteomics and phosphoproteomics data of pancreatic cancer were obtained from the CPTAC database (https://pdc.cancer.gov/pdc/). After organizing the data, we collected 211 pancreatic cancer tissue samples, consisting of 74 normal control tissues and 137 tumor tissues.

Differential expression analysis was conducted on the dataset using the ‘limma’ package in R software. The differential p-values were then subjected to False Discovery Rate (FDR) correction. We will utilize a filtering threshold of FDR values less than 0.05 to obtain differentially expressed proteins. We employed the “pheatmap” and “ggplot2” packages to generate the heatmap and volcano plot, respectively. We employ the “KSEAapp” package to conduct kinase-substrate enrichment analysis and visualize and map the resulting kinase-substrate network using Cytoscape v3.6.0 software. The analysis used R version 4.2.1 (R Foundation for Statistical Computing) [17].

### Survival analysis

Integrate the survival time and status of pancreatic cancer patients from the CPTAC database’s pancreatic cancer proteomic dataset with the protein expression data. Pancreatic cancer patients were categorized into high-expression and low-expression groups using the median protein expression with the help of the “survival” package in R. Subsequently, a survival analysis was performed [18]. *P* < 0.05 is considered statistically significant.

### Independent prognosis analysis

Integrate the survival time and status of pancreatic cancer patients from the CPTAC database’s pancreatic cancer proteomic dataset with the protein expression data. Conduct survival analysis using the “survival” package in R software, encompassing univariate and multivariate Cox analyses [18]. *P* < 0.05 is considered statistically significant.

### Pancreatic cancer classification

Using ConsensusClusterPlus package and applying consistent clustering analysis, we conducted subtype analysis on pancreatic cancer patients, evaluated the prognosis of pancreatic cancer subtypes through prognostic analysis, and performed differential analysis on proteins according to subtypes in order to obtain differential proteins in the high-risk subtypes of pancreatic cancer [16].

### Clinical relevance analysis

The pancreatic cancer proteomic dataset was used to analyze the independent prognostic-related protein expression, pancreatic cancer subtype results, and clinical characteristics of pancreatic cancer patients for clinical correlation. This analysis was performed using the ‘ComplexHeatmap’ package in the R software, creating a heatmap [19].

### GSEA analysis

The samples were grouped based on the subtypes of pancreatic cancer, and a pathway enrichment analysis (Gene Set Enrichment Analysis, GSEA) was conducted to identify differential pathway enrichment between the two groups. We used three gene sets from MSigDB to support our analysis: go.cc_analysis.Gsea.1,689,305,913,241, go.mf_analysis.Gsea.1,689,304,082,899, and kegg_analysis.Gsea.1,689,303,926,237 [20].

### GO and KEGG functional enrichment analysis

We conducted functional enrichment analysis on differentially expressed genes using the “ClusterProfiler” package in R and further visualized the enrichment results with the “ggplot2” package. We analyze cellular functions and signaling pathways primarily enriched in differentially expressed proteins, using a significance level of *P* < 0.05 as the filtering criterion [21].

### Weighted gene co-expression network analysis

The WGCNA analysis was performed using the “WGCNA” package in R software. First, the Hclust function is used for hierarchical clustering analysis. Then, the “pickSoftThreshold” function is utilized to select an appropriate soft threshold (β) and perform the adjacency matrix transformation. The Topological Overlap Matrix (TOM) is computed, and a hierarchical clustering dendrogram is constructed to divide similar protein expressions into different modules, where a minimum gene count of 50 is required. To merge potentially similar modules, a threshold of 0.25 is defined as the cutting height. Finally, the expression profiles of each module are summarized using module eigengenes (ME) and the correlation between ME and traits is calculated [19].

### Lentivirus construction

The lentiviral interference vector, pSIH1-H1-copGFP(sh-, SI501A-1 interference vector), and the lentiviral overexpression vector, pCDH-CMV-MCS-EF1α-copGFP(oe-, CD511B-1 overexpression vector), are to be purchased from System Biosciences (USA). These vectors will be used to construct a lentiviral-based interference vector for the SOX4 gene and an overexpression vector for the SOX4 and MAPK1 genes. Lentivirus particles in the lentivirus packaging kit (A35684CN, Invitrogen, USA) were successfully enclosed into HEK-293T cells (iCell-h237, Sino Biological Inc., Shanghai, China). The cell supernatant, collected after 48 h, contained the lentivirus with a 1 × 10^8^ TU/ml titer. The sequence of sh-NC is ATTCGAGGCATCACTACAGAC. The sequence of sh-SOX4-1 is AGCGACAAGATCCCTTTCATT. The sequence of sh-SOX4-2 is TGGGCACATCAAGCGACCCAT [22].

### Cell culture and grouping

Human normal pancreatic ductal epithelial cells (H6C7) sourced from Ningbo Mingzhou Biological Technology Co., Ltd. (MZ-0793) were maintained in human pancreatic epithelial cell complete growth medium (MCM-H025) also from Ningbo Mingzhou Biological Technology Co., Ltd. On the other hand, human pancreatic cancer cells PSN1 and HPAC (MZ-8139, MZ-1184) were cultured in human pancreatic cancer cell complete growth medium (CM-H153) sourced from Wuhan Puno Sai Life Science Technology Co., Ltd. The cells were divided into five groups following the experimental requirements: oe-NC, oe-SOX4, sh-NC + oe-NC, sh-SOX4 + oe-NC, and sh-SOX4 + oe-MAPK1. One mL of the appropriate lentivirus was added to the cells, and the lentiviral infection effect was examined after 48 h [23].

### RT-qPCR

Total RNA was extracted from cells using TRIzol (15,596,026, ThermoFisher, USA). The concentration and purity of the extracted total RNA were then measured using a Nanodrop 2000 micro UV spectrophotometer (ThermoFisher, USA). mRNA was reverse transcribed into cDNA using the PrimeScript RT reagent Kit (Takara Code: RR047A, Takara, Japan), and gene primers were synthesized by TaKaRa company (Table S1). Real-time fluorescence quantitative PCR detection was conducted using the 7500 Fast Real-Time PCR System (4,351,106, ThermoFisher, USA). The reaction conditions consisted of an initial denaturation at 95 °C for 10 min, followed by denaturation at 95 °C for 10 s, annealing at 60 °C for 20 s, extension at 72 °C for 34 s, and a total of 40 cycles. The relative quantification method (2^-ΔΔCT^ method) was employed to determine the relative transcription levels of the target gene, with GAPDH serving as the reference gene. ΔΔCT is calculated as the difference between ΔCT of the experimental group and ΔCT of the control group. ΔCT represents the target gene’s Ct value minus the reference gene’s Ct value. The relative transcription level of the target gene mRNA is then calculated as 2^-ΔΔCT^ [24]. Each experiment is repeated three times.

### Dual-luciferase assay

The oe-NC and oe-SOX4 vectors were separately co-transfected with a dual luciferase reporter gene vector containing the MAPK1 promoter sequence (GAACAAAGAG) and the mutant variant (CTTAGCCAGT) with a mutated MAPK1 binding site (designated as MAPK1-WT and MAPK1-MUT, respectively) into HEK293T human embryonic kidney cells to evaluate the influence of SOX4 on the transcriptional activity of the MAPK1 promoter. The expression of the internal reference gene, α-galactosidase, is utilized to compare the activation level of the target reporter gene. The cells that had been transfected were collected and lysed 48 h after transfection. The luciferase reporter gene was then detected using a luciferase assay kit (K801-200, BioVision, USA). Furthermore, the Promega dual-luciferase reporter gene analysis system (Madison, USA) was employed to detect luciferase enzymes. The activity level of the target reporter gene is determined by comparing the ratio between the relative luminescence units (RLU) obtained from the firefly luciferase assay and the RLU obtained from the Renilla luciferase assay [25].

### ChIP experiment

To evaluate the enrichment of SOX4 in the promoter region of the MAPK1 gene, we utilized the ChIP assay kit (KT101-02, Sai Cheng Biotechnology Co., Ltd., Guangzhou, China). The specific steps are as follows: When the cells reach a fusion degree of 70–80%, add 1% formaldehyde and fix them at room temperature for 10 min to crosslink and stabilize the DNA and proteins within the cells. After completing the cross-linking process, ultrasonic treatment achieves random fragmentation. Each 10-second ultrasonic treatment is followed by a 10-second interval, repeated for 15 cycles. This process results in the DNA being fragmented into appropriately sized fragments. Next, the supernatant was centrifuged at 13,000 rpm at 4 degrees Celsius and divided into three equal portions. The supernatants were incubated overnight with the following antibodies: anti-RNA polymerase II rabbit polyclonal antibody (1:100, ab238146, Abcam, UK) as a positive control, rabbit anti-IgG antibody (1:100, ab172730, Abcam, UK) as a negative control, and rabbit anti-SOX4 antibody (1:100, NBP3-18643, novusbio) for detecting the target protein. Endogenous DNA-protein complexes were precipitated by utilizing Protein Agarose/Sepharose. The supernatant was subsequently removed through brief centrifugation, followed by washing of non-specific complexes. For decrosslinking, overnight incubation at 65 degrees Celsius was conducted. Finally, the Phenol/Chloroform method was employed to extract, purify, and recover the DNA fragments intended for qPCR detection of the promoter region of the MAPK1 gene. Specific primers for the MAPK1 gene promoter region were used: Forward primer (5’-CCAGGAACATGGAGTGTGCT-3’) and Reverse primer (5’-CCAGGAACATGGAGTGTGCT-3’) [26].

### Western blot

Total protein in tissues or cells could be extracted using RIPA lysis buffer containing PMSF (P0013C, Bi Yun Tian, Shanghai, China). The sample should be incubated on ice at 4℃ for 30 min. After incubation, the supernatant should be collected by centrifugation at 8000 g for 10 min. Employ the BCA assay kit to measure the total protein concentration (23,227, ThermoFisher, USA). Fifty micrograms (50 µg) of protein were dissolved in 2× SDS sample buffer, heated for 5 min, and subjected to SDS-PAGE gel electrophoresis. Transfer the protein onto a PVDF membrane and then block it with 5% skim milk at room temperature for 1 h. Next, the PVDF membrane was incubated overnight at 4 °C with primary antibodies diluted in the following ratios: SOX4 (1:4000, NBP3-18643, novusbio), MAPK1 (1:1500, ab32081, Abcam, Cambridge, UK), IQGAP1 (1:2000, ab133490, Abcam, Cambridge, UK), p-IQGAP1 (1:1000, ab17464, Abcam, Cambridge, UK), and GAPDH (ab9485, 1:2500, Abcam, Cambridge, UK) which served as an internal control. Wash with TBST three times for 10 min each. The membrane was incubated with HRP-conjugated anti-goat IgG H&L secondary antibody (ab97051, 1:2000, Abcam, Cambridge, UK) for 1 h. Following rinsing with TBST, position the membrane onto a pristine glass slide. Take the appropriate amounts of liquids A and B, thoroughly mix them, and apply them to the membrane. The Bio-Rad image analysis system from BIO-RAD Corporation in the United States was utilized to perform the analysis. The Quantity One v4.6.2 software was employed for this purpose. The relative protein content was determined by dividing each protein band’s grayscale value by the GAPDH protein band [27]. Repeat the experiment three times and take the average.

### CCK-8 assay

The results of the cell proliferation experiment were analyzed using the CCK-8 kit (CA1210, Beijing Solaibao Technology Co., Ltd., Beijing, China), and the statistical analysis was performed. Cells from the logarithmic growth phase were plated at a density of 1 × 10^4^ cells per well in a 96-well plate for pre-culture for 24 h. Following pre-culture, the cells were transfected based on the assigned groups. After a 48-hour transfection, 10 µL of CCK-8 reagent was added at 0, 24, 48, and 72 h post-transfection. The cells were then incubated at 37℃ for 3 h. The absorbance value of each well at a wavelength of 450 nm should be measured using a spectrophotometer. This value is directly related to cell proliferation in the culture medium and will be used to generate the cell growth curve [28].

### Cloning formation experiment

Use cloning formation experiments to determine cell proliferation. Inoculate the cells at a density of 2000 cells per dish in a 6 cm culture dish. Replace the culture medium with fresh medium, and after 14 days of cell growth, the colonies were stained using a 0.5% weight/volume solution of crystal violet (C8470, Soleibao Technology Co., Ltd., Beijing, China) to facilitate photography and quantitative observation of colony formation. A clone could be defined as a formed cell cluster containing more than 50 cells [23].

### Transwell experiment

For the invasion experiment, the Matrigel gel (356,234, Shanghai Haoyang Biotechnology Co., Ltd., Shanghai, China) stored at -80℃ should be retrieved and left to equilibrate at 4℃ overnight to thaw into a liquid state. Add 200 µL of Matrigel gel to 200 µL of serum-free culture medium at 4℃. Mix thoroughly to dilute the gel matrix. Then, add 50 µL of the diluted gel to the upper chamber of each Transwell plate and place them in the incubator. Wait for gel solidification, which typically takes 2–3 h. The cells were digested and counted, and a cell suspension was prepared using a serum-free culture medium. 200 µL of cell suspension was added to each upper chamber, while the lower chamber was filled with 800 µL of conditioned medium containing 20% FBS. Incubate for 24 h in a 37℃ incubator. Remove the Transwell plate from the experiment, rinse it two times with phosphate-buffered saline (PBS), and gently wipe the top surface of the cells using a cotton ball. Next, the specimen was fixed with formaldehyde for 10 min, followed by three thorough rinses with water. The sample was stained using a 0.1% Crystal Violet solution and incubated at room temperature for 30 min. After incubation, the sample was rinsed twice with PBS. Observe, photograph, and count using an inverted microscope. Migration experiments do not necessitate extracellular matrix gel coating, and the incubation duration is 24 h [29]. A minimum of four cells should be counted under the microscope in randomly selected fields of view. The experiment is repeated three times.

### Nude mouse pancreatic cancer model

Fifty-four BALB/c nude mice, aged 6 weeks, were obtained from Beijing Vital River Laboratory Animal Technology Co., Ltd., located in Beijing, China. The nude mice are individually housed in separate cages within an SPF-grade animal laboratory. The laboratory maintains a humidity level of 60–65% and a temperature range of 22 to 25℃. The experiment began after one week of adaptive feeding, during which the health condition of the hairless mice was observed. The Animal Ethics Committee of our institution has approved the experimental protocol and procedures for animal use.

In this study, nude mice were utilized to establish subcutaneous and orthotopic pancreatic cancer xenograft models and a liver metastasis model in nude mice. The models are divided into the sh-NC + oe-NC group, the sh-SOX4 + oe-NC group, and the sh-SOX4 + oe-MAPK1 group. Each group consists of six nude mice.

A subcutaneous xenograft model of pancreatic cancer in nude mice was established using PSN1 pancreatic cancer cell lines. The cell lines were prepared as a cell suspension with a concentration of 2 × 10^7^ cells/mL in each group. Next, using a 1 mL syringe, draw 0.2 mL of the cell suspension for injection and subcutaneously inject it into the nude mouse’s left axillary region. After vaccination, it is important to continue housing all nude mice in a facility with SPF-grade conditions. On the 36th day, euthanasia was conducted on the nude mice in each group by dislocating their cervical vertebrae. Subsequently, tumor tissues were extracted and weighed while concurrently documenting lymph node metastasis in the armpits of each group of nude mice.

An in-situ xenograft model of pancreatic cancer was established in nude mice. 200 µL of PSN1-lucifer cells (2 × 10^7^) (L7840, Beijing Solaibao Technology Co., Ltd., Beijing, China) were injected into the pancreatic tissue of the nude mice. Four weeks after injection, the nude mice were transferred to the imaging room and subjected to imaging using the IVIS Lumina XR instrument (PerkinElmer, Waltham, MA, USA) to capture both white light and bioluminescence images.

Nude mouse liver metastasis model: Each group of PSN1 cells (2 × 10^7^) was intravenously injected into the nude mice using a 0.2 mL cell suspension. Thirty-six days later, each group of nude mice was euthanized using the cervical dislocation method. Liver tissues were extracted, and H&E staining was performed to determine the average number of liver metastases [23].

### H&E staining

The liver tissue slices from the naked mole rat liver metastasis model should be flattened and then dewaxed in water. Next, the liver tissue sections were stained using the hematoxylin and eosin (HE) staining reagent kit (PT001, Shanghai Bogu Biological Technology Co., Ltd., Shanghai, China) per the manufacturer’s instructions. The main steps are as follows: Sudan III staining is performed at room temperature for 10 min, followed by rinsing with water for 30–60 s. Then, 1% hydrochloric acid alcohol differentiation is done for 30 s, then rinsing with water and soaking for 5 min. Next, Eosin staining is carried out at room temperature for 1 min. The tissue is dehydrated with an alcohol gradient (70%, 80%, 90%, 95%, 100%) for 1 min at each gradient. Xylene is applied for 1 min, and the tissue is then made transparent in xylene I and II, each for 1 min twice. After that, the tissue is sealed in a ventilated cabinet with neutral resin. Finally, the histological changes in each hepatic tissue group are observed and photographed using an optical microscope (BX50; Olympus Corp, Tokyo, Japan) [30].

### Statistical analysis

Statistical analysis for this study was performed using IBM’s SPSS software (version 21.0) in the United States. The measurement data are expressed as Mean ± SD. Comparisons between two data groups with a normal distribution were conducted using the unpaired Student’s t-test. Multiple group comparisons were performed using a one-way ANOVA followed by Tukey’s post hoc test.

## Results

### MAPK1 kinase activity and IQGAP1 phosphorylation as potential drivers in pancreatic cancer progression

Pancreatic ductal adenocarcinoma (PDAC) is among the most lethal solid malignancies. In recent years, proteomics and post-translational modification analysis have emerged as critical tools in cancer research. They offer substantial implications in enhancing our comprehension of tumor pathogenesis, identifying novel biomarkers and therapeutic targets, and further assisting in diagnosing and treating this disease [31]. Hence, we investigate the possible molecular mechanisms that regulate the progression of pancreatic cancer, using proteomics data of pancreatic cancer and phosphorylation.

**Fig. 1:**
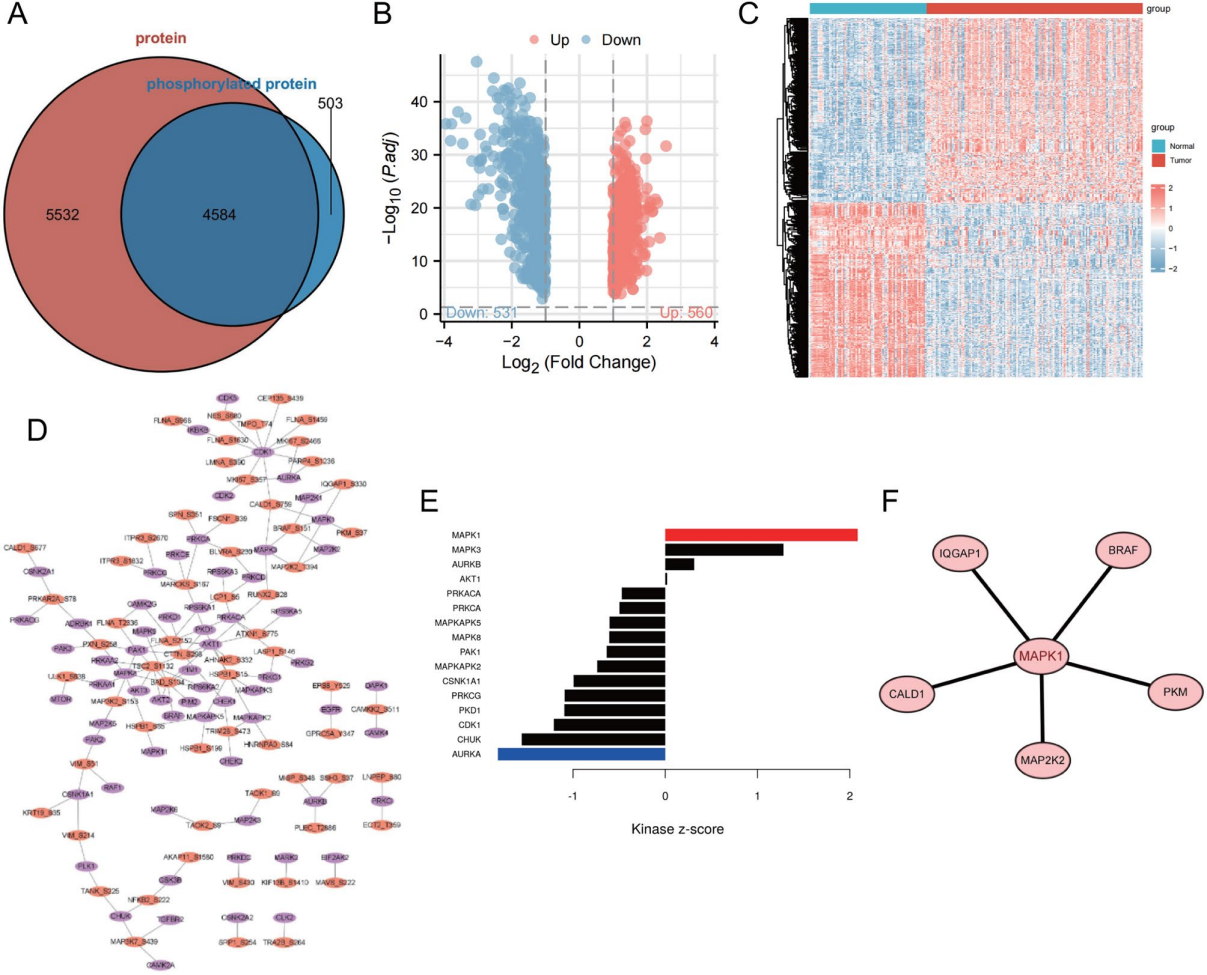
Differential Analysis and KSEA analysis of phosphoproteomic data in pancreatic cancer. *Note *(**A**) Venn diagram representing the intersection between proteins and phosphorylated proteins. (**B**) Volcano plot showing differential phosphorylation site patterns in the dataset, with red dots indicating upregulated phosphorylation levels and blue dots indicating downregulated phosphorylation levels. (**C**) Heatmap of phosphorylation levels at differential peptide phosphorylation sites in the dataset, with hierarchical clustering based on phosphorylation levels on the left dendrogram, color bar on the right representing the gradient, with red indicating high phosphorylation levels, and blue indicating low phosphorylation levels. Above the histogram, red represents pancreatic cancer tissue samples (137 cases), and blue represents normal pancreatic tissue samples (74 cases). (**D**) Kinase-substrate network in KSEA analysis, purple representing kinases and orange representing substrates. (**E**) Bar graph of KSEA analysis results, with red bars representing upregulated kinases in pancreatic cancer tissue and blue bars representing downregulated kinases in pancreatic cancer tissue, while black bars represent kinases with no difference. (**F**) Protein-protein interaction network of the substrate corresponding to MAPK1.

To begin, retrieve the pancreatic cancer proteome and phosphoproteome data from the CPTAC database. After organizing the data, we found 211 pancreatic tissue samples, consisting of 74 normal control tissues and 137 tumor tissues. The proteins detected in these tissue samples overlap with those undergoing phosphorylation modification, as depicted in Fig. 1A. A total of 10,116 proteins were detected, of which 4,584 were observed to undergo phosphorylation modification.

Subsequently, differential analysis was conducted on the phosphorylation sites of the peptide fragments. The results (Fig. 1B-C) indicated an upregulation in the phosphorylation levels of 560 phosphorylation sites in pancreatic cancer tissues. We conducted a Kinase-Substrate Enrichment Analysis (KSEA) on the phosphorylation sites of peptides that were upregulated based on the phosphorylation level. The results (Fig. 1D-E) revealed that in pancreatic cancer tissues, the kinase activity of MAPK1 was higher than in the normal control group. The kinase MAPK1 had several substrates: BRAF, IQGAP1, CALD1, MAP2K2, and PKM (Fig. 1F).

**Fig. 2:**
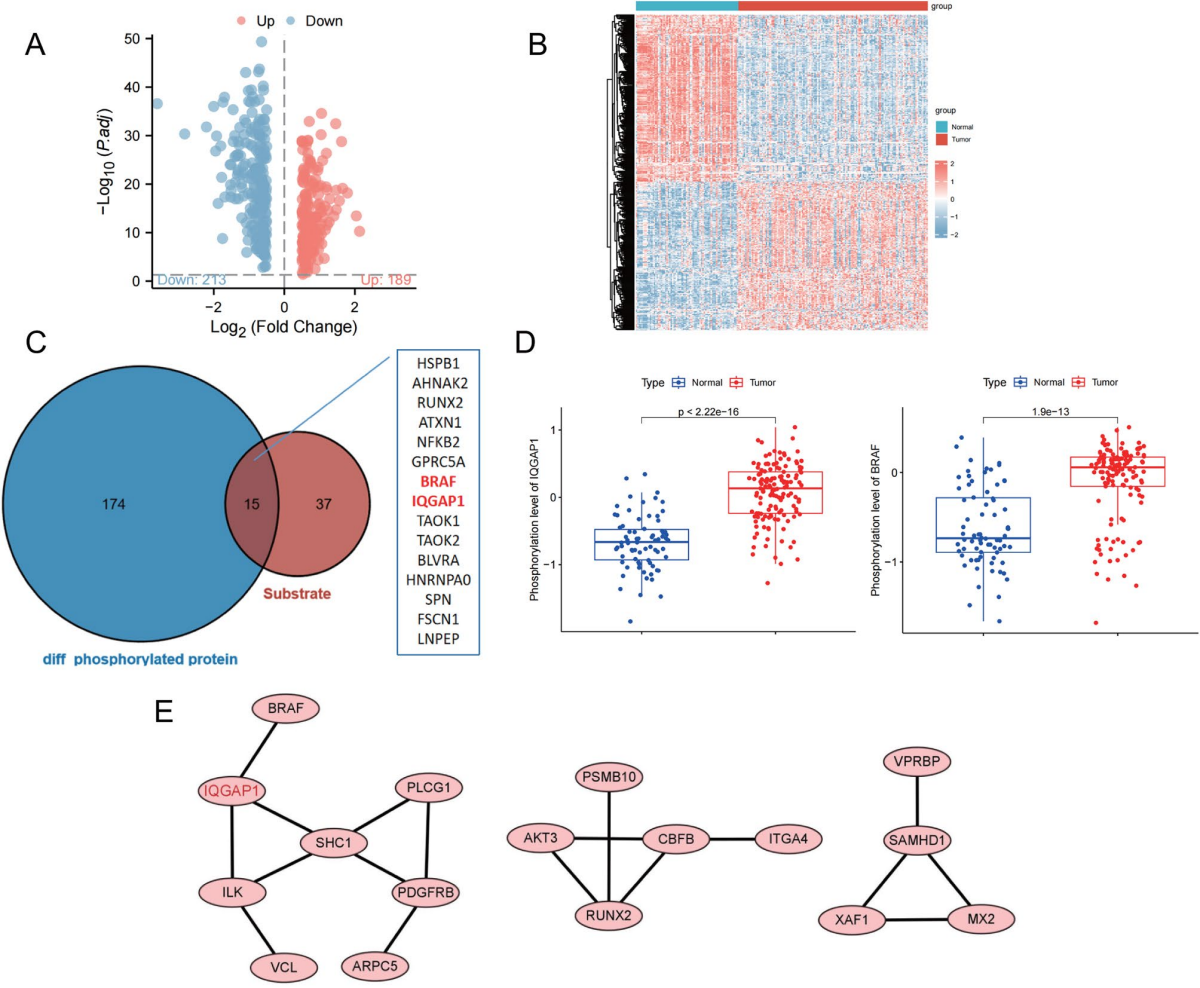
Potential Impact of MAPK1-Mediated Phosphorylation of IQGAP1 on the Development of Pancreatic Cancer. *Note *(**A**) Volcano plot of differentially phosphorylated peptide-derived proteins in the dataset, with red dots indicating upregulated expression and blue dots indicating downregulated expression. (**B**) Heatmap of differential protein phosphorylation levels in the dataset, with hierarchical clustering based on protein phosphorylation levels on the left dendrogram, color bar on the right representing the gradient, with red indicating high phosphorylation levels and blue indicating low phosphorylation levels. Above the histogram, blue represents cancer tissue samples in the C1 subtype (61 cases), and red represents cancer tissue samples in the C2 subtype (73 cases). (**C**) Venn diagram representing the intersection between substrates and upregulated phosphorylated proteins in KSEA analysis. (**D**) Bar graph of BRAF and IQGAP1 protein phosphorylation levels in the dataset, with red representing pancreatic cancer tissue samples (137 cases) and blue representing normal pancreatic tissue samples (74 cases). (**E**) PPI network is constructed based on upregulated phosphorylated proteins

Moreover, we performed differential analysis on proteins sourced from phosphorylated peptides, revealing (Fig. 2A-B) a total of 189 proteins exhibiting elevated phosphorylation levels in pancreatic cancer tissues. The intersection of substrates and phosphorylation levels that were upregulated in KSEA analysis revealed (Fig. 2C-D) that the phosphorylation levels of BRAF and IQGAP1 proteins were significantly increased in pancreatic cancer tissues. Previous studies have shown that phosphorylation modifications can affect protein-protein interactions, and through PPI networks, it is possible to understand the important regulatory role of phosphorylated proteins [32]. Building on the upregulated phosphorylation levels of proteins, a PPI network was constructed, and it was found (Fig. 2E) that the interaction between IQGAP1 and other proteins was more significant compared to BRAF. Therefore, we speculate that in pancreatic cancer, the kinase MAPK1 may play an important role in tumor progression by promoting the phosphorylation modification of IQGAP1.

### Identification and prognostic analysis of differentially expressed proteins in pancreatic Cancer reveals two patient subtypes

**Fig. 3:**
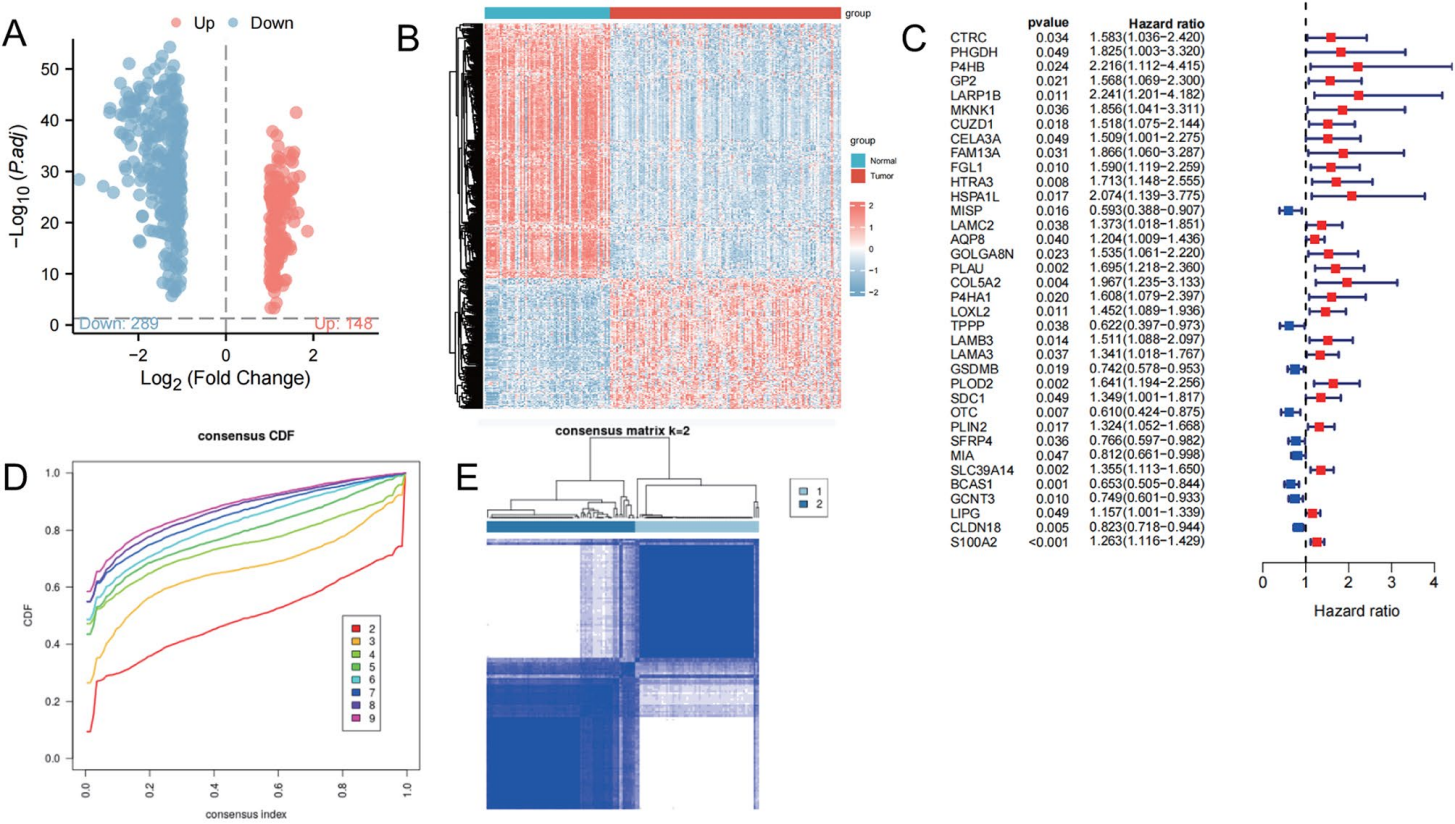
Subtyping of Pancreatic Cancer Patients. *Note *(**A**) Volcano plot of differential protein expression in the dataset, with red dots indicating upregulated expression and blue dots indicating downregulated expression. (**B**) Heatmap of differential protein expression levels in the dataset, with hierarchical clustering based on protein expression levels on the left dendrogram, color bar on the right representing the gradient, red indicating upregulated expression and blue indicating downregulated expression. Above the histogram, red represents pancreatic cancer tissue samples (137 cases), and blue represents normal pancreatic tissue samples (74 cases). (**C**) Forest plot of independent prognosis analysis, with blue dots representing low-risk proteins and red dots representing high-risk proteins. (**D**) Curve graph evaluating the subtypes of pancreatic cancer, with different colored curves representing the number of subtypes. (**E**) Cluster map of pancreatic cancer subtypes, with 61 cases in the C1 subtype and 73 cases in the C2 subtype, originally 137 pancreatic cancer patients, with 3 cases deleted due to missing survival information

Subsequently, we conducted a differential analysis of the total proteins in pancreatic cancer using proteomic data. The analysis revealed 437 differentially expressed proteins in pancreatic cancer tissues, with 148 proteins upregulated and 289 downregulated. These results are presented in Fig. 3A-B. Further analysis of 437 differentially expressed proteins was conducted for prognosis, resulting in a total of 58 prognostic-associated proteins (Table S1). After independent prognosis analysis, a final selection of 36 proteins was made (Fig. 3C). Based on these 36 proteins, pancreatic cancer patients were divided into two subtypes, C1 subtype and C2 subtype, using consistent clustering analysis (Fig. 3D-E). In conclusion, pancreatic cancer patients can be classified into two subtypes based on the analysis of 36 independently prognostic-related proteins.

### Correlation of pancreatic cancer subtypes with prognosis and tumor malignancy reveals high-risk C2 subtype with aberrant pathway activation

**Fig. 4:**
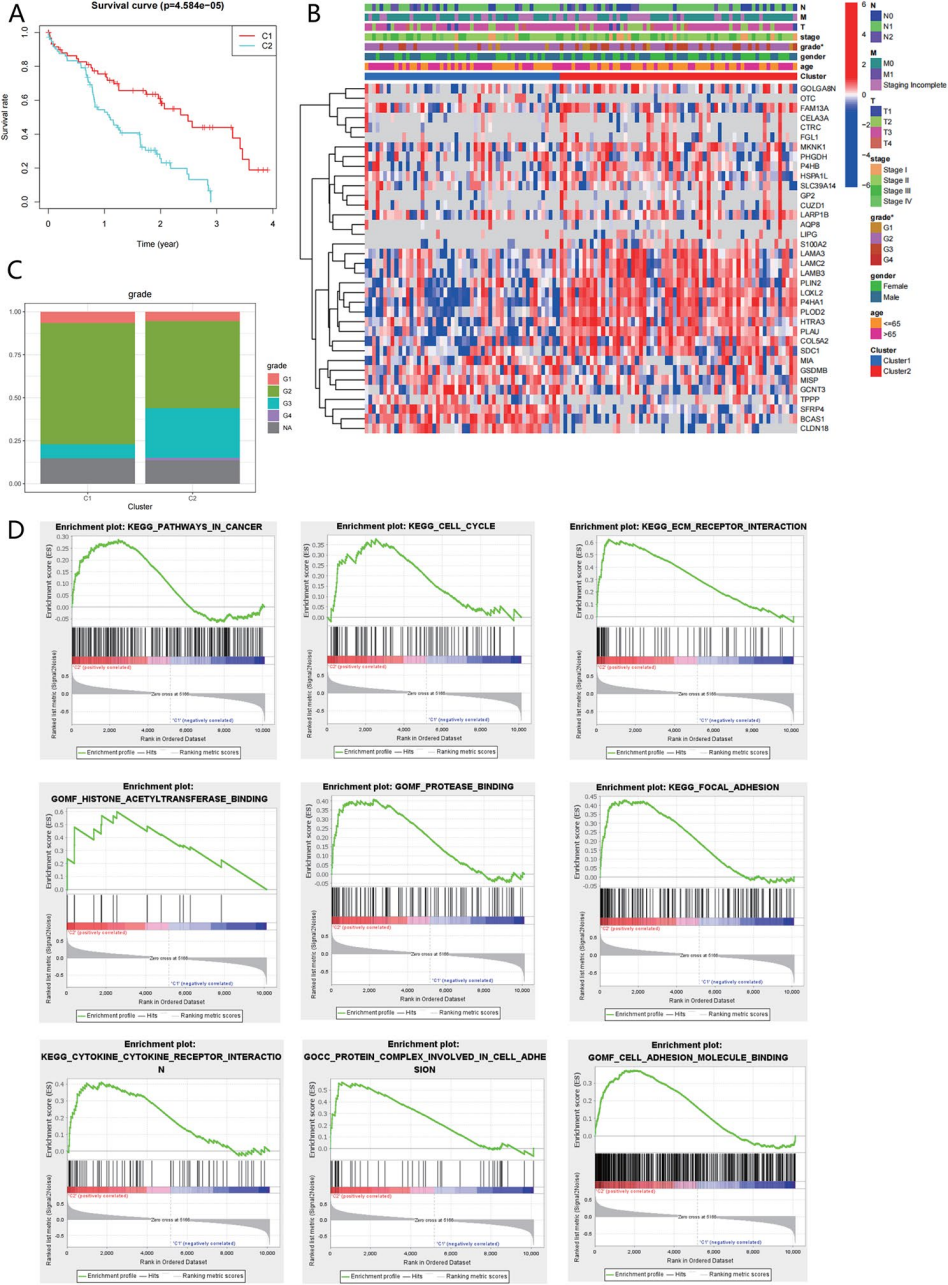
Prognosis and GSEA Analysis of Pancreatic Cancer C1/C2 Subtypes. *Note *(**A**) Prognosis analysis of pancreatic cancer C1/C2 subtypes, with red representing the C1 subtype (61 cases) and blue representing the C2 subtype (73 cases). (**B**) Heatmap of clinical relevance with pancreatic cancer C1/C2 subtypes, with a color bar on the top indicating different clinical characteristics. The asterisk (*) indicates significance at *p* < 0.05. (**C**) Percentage of clinical grade in C1/C2 subtypes. (**D**) Enriched pathways in GSEA analysis for C1/C2 subtypes

The previous analysis divided patients with pancreatic cancer into two subtypes. Then, a correlation analysis of prognosis and clinical characteristics was conducted on the C1/C2 subtypes. The results demonstrated that patients with the C2 subtype had a worse prognosis than those with the C1 subtype (Fig. 4A). Additionally, there was a correlation between the C2 subtype and tumor malignancy grading (Fig. 4B-C). These findings suggest that the C2 subtype represents a high-risk group among pancreatic cancer patients.

Furthermore, an enrichment analysis using GSEA was performed to identify the enriched pathways in the C1/C2 subtypes. As depicted in Fig. 4D, the results revealed that the C2 subtype exhibited aberrant activation of numerous tumor-related pathways. In conclusion, pancreatic cancer patients with the high-risk C2 subtype are linked to a poor prognosis and aggressive tumor subtyping, and there is also a high abundance of tumor-related pathways.

### Role of protein phosphorylation in distinguishing high-risk C2 subtype: implications for IQGAP1 and tumor-related pathways in pancreatic cancer progression

**Fig. 5:**
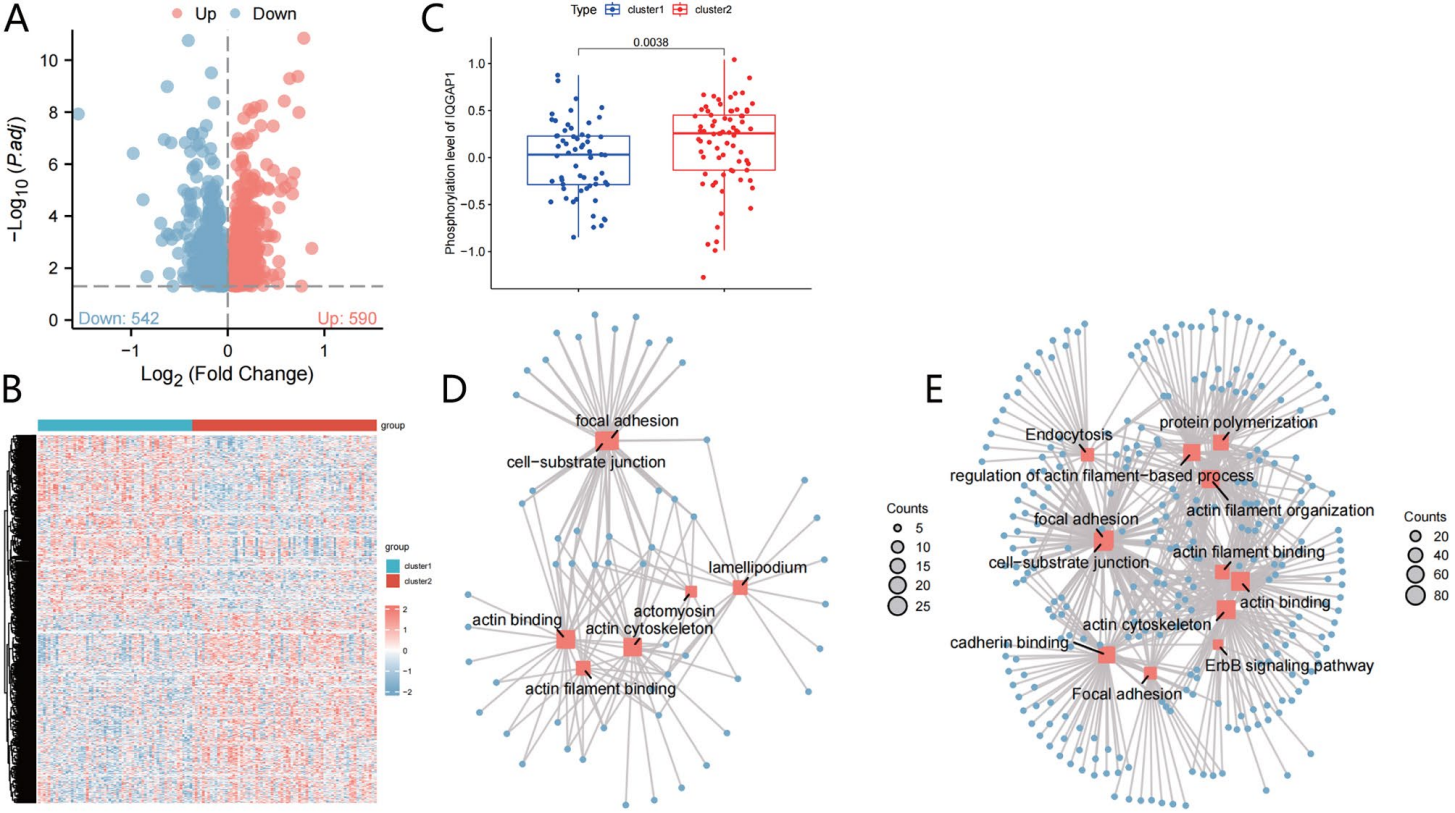
Differential Analysis of Phosphorylated Proteins and Pathway Enrichment Analysis in Pancreatic Cancer Patients’ C1/C2 Subtypes. *Note *(**A**) Volcano plot of differentially phosphorylated proteins in pancreatic cancer patients’ C1/C2 subtypes. Red dots represent upregulated phosphorylation levels; blue dots represent downregulated phosphorylation levels; (**B**) Heatmap of phosphorylation levels of differentially expressed proteins in pancreatic cancer patients’ C1/C2 subtypes. The dendrogram on the left is based on protein phosphorylation levels; the color bar on the right represents the intensity of phosphorylation, with red indicating high phosphorylation levels and blue indicating low phosphorylation levels. The histogram at the top represents C1 subtype cancer tissue samples (61 cases) in blue and C2 subtype cancer tissue samples (73 cases) in red; (**C**) Bar chart showing the phosphorylation levels of IQGAP1 protein in pancreatic cancer patients’ C1/C2 subtypes, with blue representing C1 subtype (61 cases) and red representing C2 subtype (73 cases); (**D**) Network of pathway enrichment analysis for upregulated phosphorylated proteins in pancreatic cancer tissues. Red dots represent enriched pathway names, the size of the dots represents the number of enriched proteins, and the larger the dot, the greater the number of enriched proteins. Blue dots represent the enriched proteins; (E) Network of pathway enrichment analysis for upregulated phosphorylated proteins in pancreatic cancer tissues of the C2 subtype

To further analyze and reveal the overall situation of protein phosphorylation modifications in the C1/C2 subtypes, we conducted differential analysis of phosphorylated proteins, and the results showed (Fig. 5A-B): compared to the C1 subtype, there were a total of 590 significantly upregulated phosphorylation levels of proteins in the C2 subtype. Previous studies have revealed that the phosphorylation level of IQGAP1 protein is significantly elevated in pancreatic cancer tissues (Fig. 2D). This is consistent with the significant increase trend of IQGAP1 protein phosphorylation level in the C2 subtype (Fig. 5C), indicating that phosphorylation modification of IQGAP1 protein may promote the development of high-risk C2 subtype and play an important role in the progression of pancreatic cancer.

Furthermore, the pathway enrichment analysis of the results revealed that the phosphorylation levels of proteins enriched in tumor-related pathways (e.g., Focal adhesion, cell-substrate junction, actin filament binding) were upregulated in the C2 subtype compared to the C1 subtype (Fig. 5D-E). It aligns with the pathway enrichment observed in phosphorylated proteins between tumor and normal tissue. Research has shown that phosphorylation of the proteins involved in these pathways plays a crucial role in developing and advancing pancreatic cancer. Previous studies have shown that IQGAP1 functions as a cellular scaffold regulator by interacting with multiple proteins and participating in diverse biological activities, such as regulating cell cytoskeleton dynamics, cell-cell adhesion, cell invasion, and proliferation, and thereby exerting oncogenic effects [33–35].

In conclusion, the phosphorylation modification of the IQGAP1 protein may promote the occurrence of the high-risk C2 subtype in pancreatic cancer and play a crucial role in the initiation and progression of pancreatic cancer.

### Unveiling the regulatory role of transcription factor SOX4 in high-risk C2 subtype of pancreatic cancer: implications for MAPK1 and IQGAP1 modulation

**Fig. 6:**
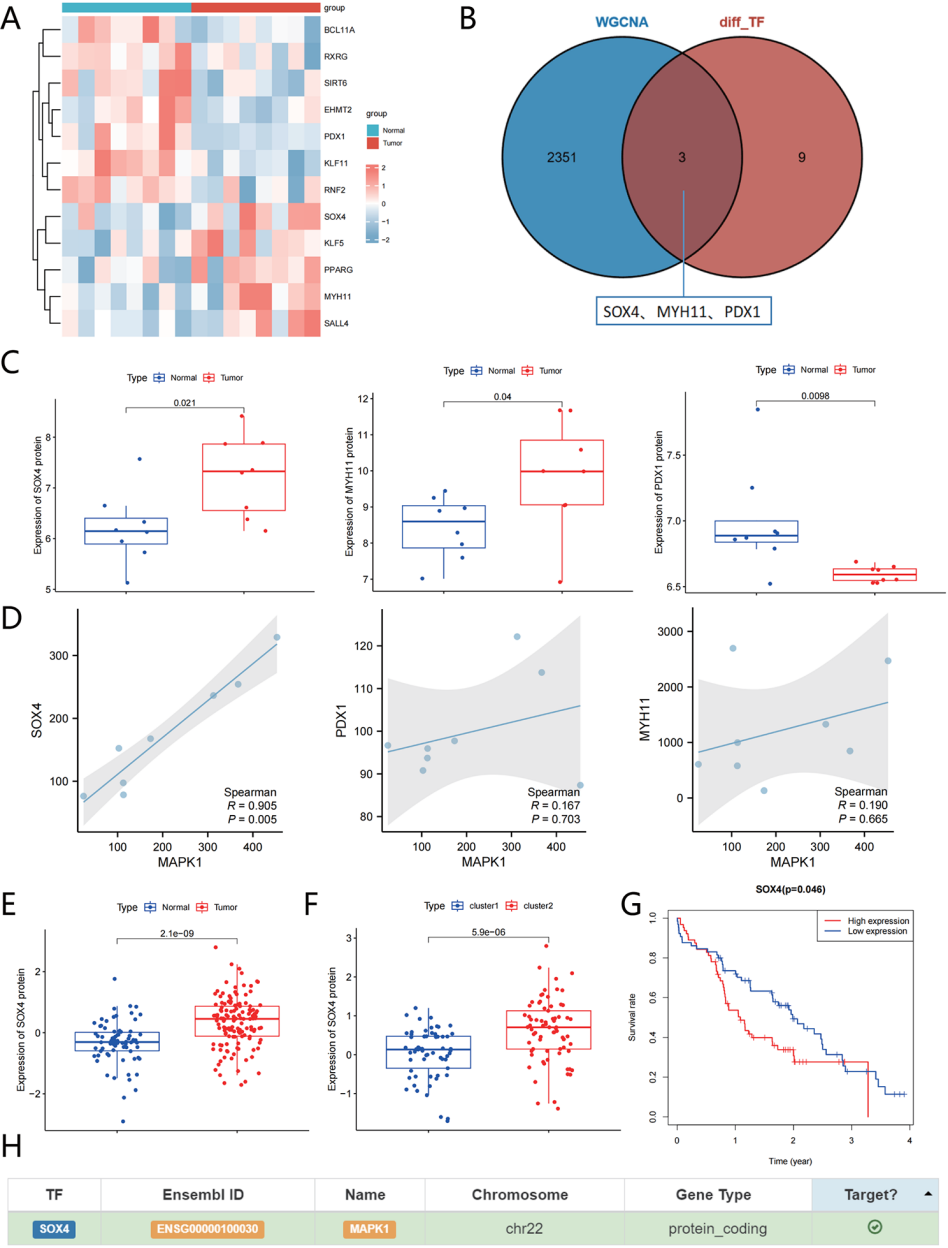
Predicted molecular mechanism of SOX4-MAPK1-IQGAP1 phosphorylation modification. *Note *(**A**) Heatmap of differentially expressed tumor-related transcription factors in pancreatic cancer tissue proteomic sequencing data. The dendrogram on the left is based on protein expression levels; the color bar on the right represents the intensity of expression, with red indicating upregulated expression and blue indicating downregulated expression. The histogram at the top represents normal adjacent tissue samples (8 cases) in blue and pancreatic cancer tissue samples (8 cases) in red; (**B**) Venn diagram showing the intersection of 12 differentially expressed tumor-related transcription factors in pancreatic cancer tissues and proteins enriched in the turquoise module; (**C**) Bar chart showing the expression of SOX4, MYH11, and PDX1 proteins in pancreatic cancer tissue proteomic sequencing data, with blue representing normal adjacent tissue samples (8 cases) and red representing pancreatic cancer tissue samples (8 cases); (**D**) Analysis of the correlation between SOX4, MYH11, PDX1 proteins, and kinase MAPK1 in pancreatic cancer tissue proteomic sequencing data; (**E**) Bar chart showing the expression of SOX4 protein in pancreatic cancer-related proteomic data from the CPTAC database, with red representing pancreatic cancer tissue samples (137 cases) and blue representing normal pancreatic tissue samples (74 cases); (**F**) Bar chart showing the expression of SOX4 protein in pancreatic cancer patients’ C1/C2 subtypes, with blue representing C1 subtype (61 cases) and red representing C2 subtype (73 cases); (**G**) Prognostic analysis of pancreatic cancer patients based on the median expression of SOX4 protein, with blue representing the high expression group (67 cases) and red representing the low expression group (67 cases); (**H**) Predicted diagram of SOX4 involvement in the regulation of MAPK1 transcription

In order to investigate and reveal the potential molecular mechanisms regulating the progression of pancreatic cancer from multiple aspects, we performed protein sequencing on 8 pancreatic cancer tissues and adjacent tissues. The expression level of proteins is generally regulated by transcription factors at the transcriptional level. In order to identify the upstream transcription factors regulating the kinase MAPK1, we extracted tumor-related transcription factors from the sequencing data and differential analysis showed (Fig. 6A): a total of 12 tumor-related transcription factors were differentially expressed in pancreatic cancer tissues.

WGCNA analysis was performed on the pancreatic cancer-related total proteins in the CPTAC database. The results showed that when the scale-free fit index r2 = 0.9, the minimum soft threshold β = 2, which meets the criteria for constructing a scale-free network. Therefore, we chose r2 = 0.9 and β = 2 as the standards for WGCNA module identification (Figure S1A). Four co-expression modules were identified in the expression matrix, among which the turquoise module showed the most significant positive correlation with the disease status (Figure S1B). Therefore, we intersected the 12 tumor-related transcription factors differentially expressed in pancreatic cancer tissues with the proteins enriched in the turquoise module, and obtained three proteins, SOX4, MYH11, and PDX1 (Fig. 6B). Among them, SOX4 and MYH11 were significantly upregulated, while PDX1 was significantly downregulated (Fig. 6C). Moreover, only the protein SOX4 showed a significant positive correlation with the kinase MAPK1 (Fig. 6D). Previous studies have shown that SOX4 is involved in promoting tumor metastasis [36, 37]. Therefore, we selected SOX4 protein for further study in pancreatic cancer tissues.

The protein data associated with pancreatic cancer in the CPTAC database (Fig. 6E-F) demonstrates a high expression of SOX4 in pancreatic cancer tissues, with an upregulation observed in the C2 subtype compared to the C1 subtype. The overexpression of the SOX4 protein is strongly correlated with a negative prognosis in patients with pancreatic cancer (Fig. 6G). The online platform predicted that SOX4 potentially participates in the regulation of MAPK1 transcription, as indicated in Fig. 6H.

In conclusion, the transcription factor SOX4 may promote the development of the high-risk C2 subtype of pancreatic cancer by regulating the transcription of MAPK1 and promoting the phosphorylation modification of IQGAP1. It plays a critical role in the occurrence and progression of pancreatic cancer.

### Experimental validation of SOX4-MAPK1-IQGAP1 Axis: unraveling the regulatory mechanisms in pancreatic cancer progression

**Fig. 7:**
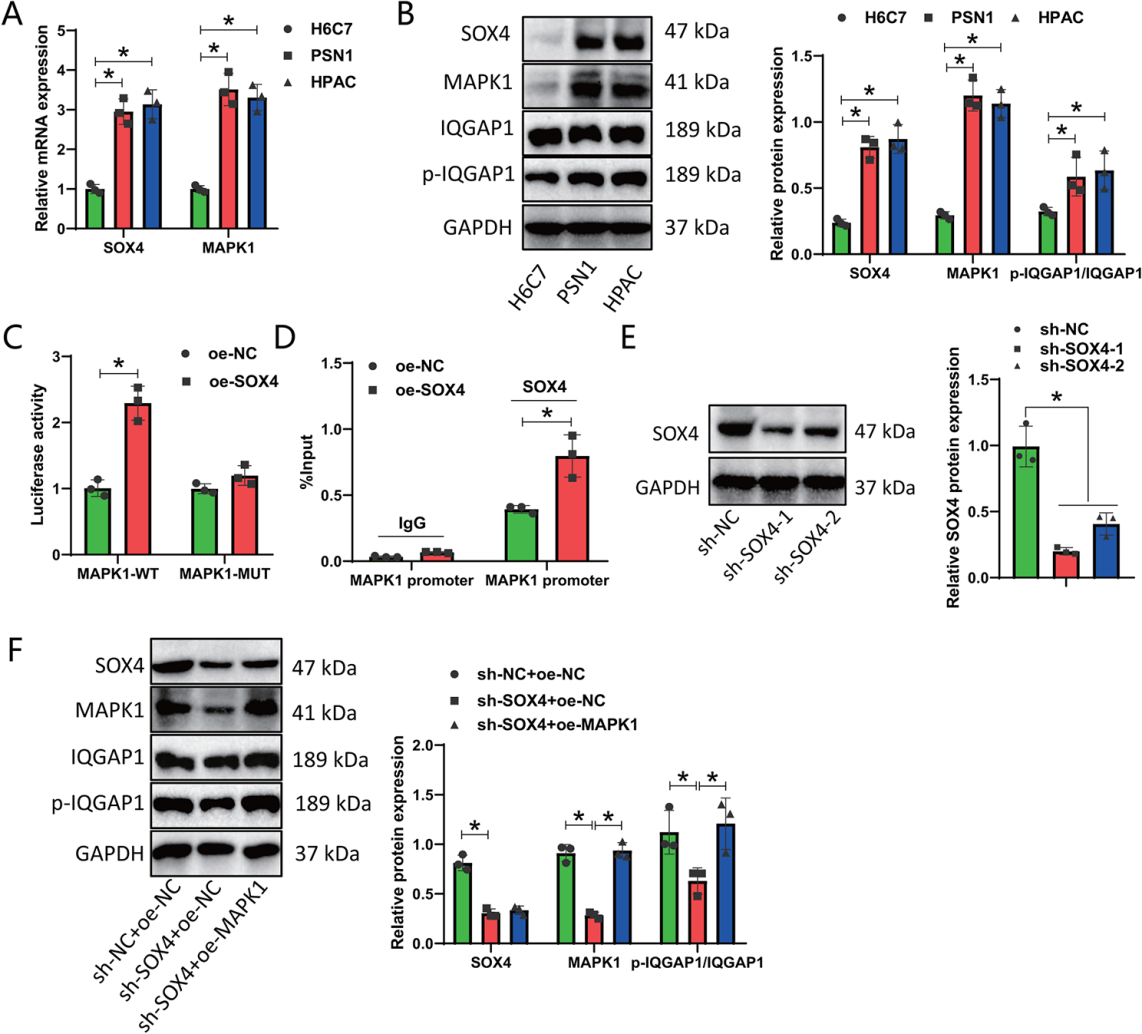
Molecular mechanism of SOX4-MAPK1-IQGAP1 phosphorylation regulation. *Note *(**A**) RT-qPCR analysis of SOX4 and MAPK1 mRNA expression levels in pancreatic cancer cells; (**B**) Western blot analysis of SOX4, MAPK1 protein expression levels, and IQGAP1 phosphorylation levels in pancreatic cancer cells; (**C**) Dual luciferase assay to assess the effect of SOX4 on MAPK1 promoter activity; (**D**) ChIP assay to detect the enrichment of SOX4 on the MAPK1 promoter; (**E**) Western blot analysis of siRNA efficiency in silencing SOX4; (**F**) Western blot analysis of SOX4, MAPK1 protein expression levels, and IQGAP1 phosphorylation levels in different groups of pancreatic cancer cells. *P < 0.05 compared to control group. All cell experiments were performed in triplicate

Based on the bioinformatics analysis above, we speculate that in pancreatic cancer, the transcription factor SOX4 may be involved in promoting the phosphorylation modification of IQGAP1 by regulating the transcription of MAPK1. Next, we will further validate this in vitro cell experiment. First, the expression levels of SOX4, MAPK1, and phosphorylated IQGAP1 in pancreatic cancer cells were detected using RT-qPCR and Western blot (Fig. 7A-B). The results showed that compared to the normal human pancreatic ductal epithelial cells (H6C7), the expression levels of SOX4 and MAPK1, as well as the phosphorylation level of IQGAP1, were significantly increased in pancreatic cancer cells. The impact of SOX4 on the promoter activity of MAPK1 was tested using a dual-luciferase reporter assay, and the results showed that in the co-transfection group with MAPK1-WT, the overexpression of SOX4 significantly increased the promoter activity of MAPK1; while in the co-transfection group with MAPK1-MUT, the overexpression of SOX4 had no significant effect on the promoter activity of MAPK1 (Fig. 7C). The ChIP assay results demonstrated that after overexpressing SOX4, there was a significant increase in the enrichment of SOX4 on the MAPK1 promoter (Fig. 7D), indicating that SOX4 can promote the transcription of MAPK1 by binding to the MAPK1 promoter.

Next, pancreatic cancer cells HPAC were grouped and treated. The Western blot results showed that after silencing SOX4, the expression of SOX4 significantly decreased (sh-SOX4-1 showed better silencing effect and was selected for subsequent experiments), and the expression levels of MAPK1 and the phosphorylation level of IQGAP1 were significantly reduced. Meanwhile, after overexpressing MAPK1, the expression levels of MAPK1 and the phosphorylation level of IQGAP1 were reversed (Fig. 7E-F). This indicates that the phosphorylation level of IQGAP1 can be regulated by the kinase MAPK1, and overexpression of MAPK1 can promote the phosphorylation of IQGAP1.

In summary, the activation of MAPK1 transcription by SOX4 promotes the phosphorylation modification of IQGAP1.

### Functional assays reveal SOX4 as a key regulator of pancreatic cancer cell proliferation, migration, and invasion via MAPK1-IQGAP1 phosphorylation

**Fig. 8:**
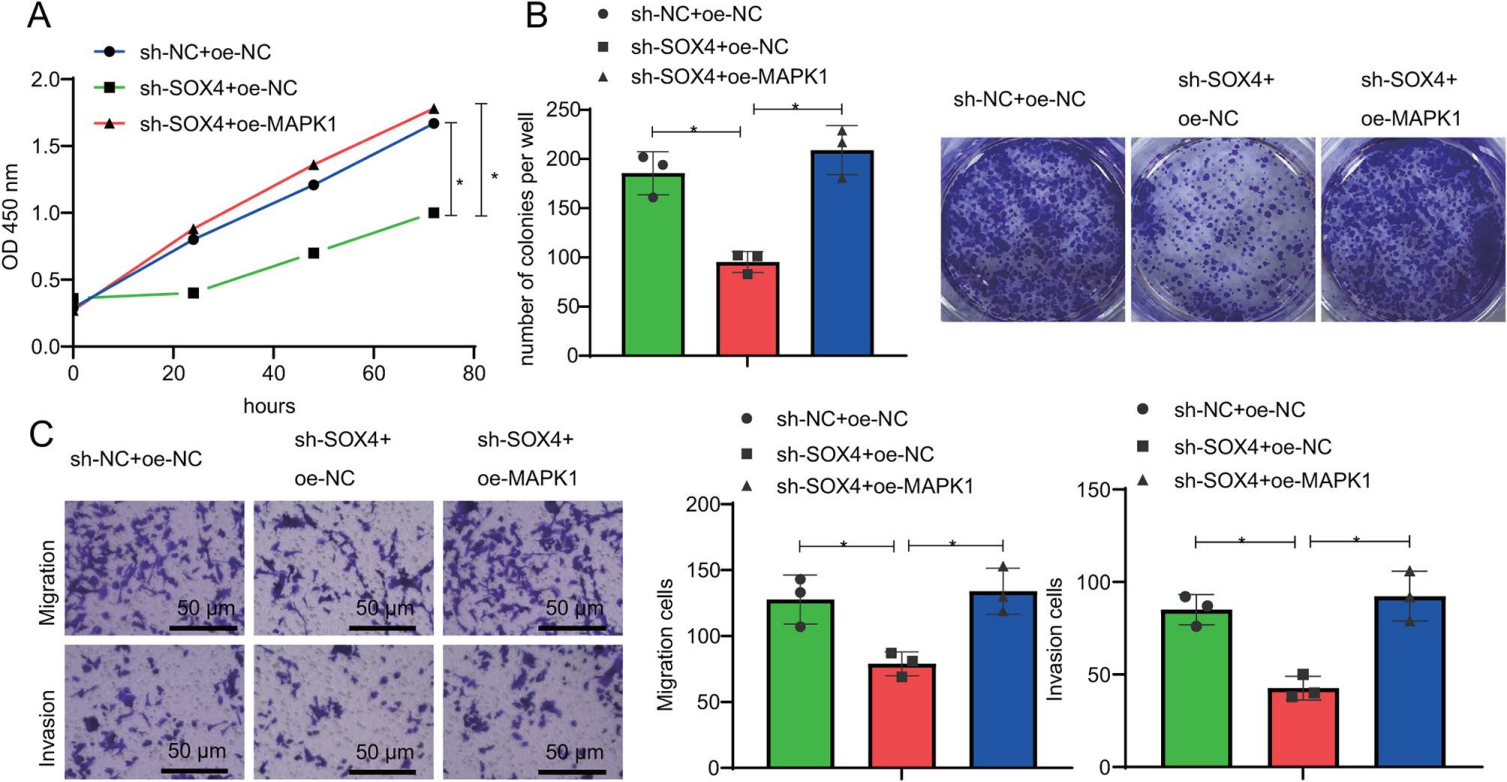
The impact of SOX4 on proliferation, migration, and invasion of PSN1 pancreatic cancer cells through regulation of MAPK1-IQGAP1 phosphorylation. *Note *(**A**) CCK-8 assay was performed to evaluate the proliferation of PSN1 cells in different groups; (**B**) Clonogenic assay was conducted to assess the colony-forming ability of PSN1 cells in different groups; (**C**) Transwell assay was employed to measure the migration and invasion capabilities of PSN1 cells in different groups, Scale bar = 200 μm. * indicates a difference (*P* < 0.05) between the two groups, and all cell experiments were repeated three times

To evaluate the effects of SOX4 on the biological functions of pancreatic cancer cells by activating the MAPK1 transcription and promoting IQGAP1 phosphorylation modification, we conducted functional tests on grouped pancreatic cancer cells using CCK-8, colony formation, and Transwell experiments. The results showed (Fig. 8A-C; Figure S2A-C): after silencing SOX4, the proliferation, migration, and invasion abilities of pancreatic cancer cells were significantly reduced; meanwhile, overexpression of MAPK1 reversed the proliferation, migration, and invasion abilities of pancreatic cancer cells. This indicates that silencing SOX4 can suppress the proliferation, migration, and invasion of pancreatic cancer cells by reducing the MAPK1-IQGAP1 phosphorylation level.

### In vivo validation of SOX4’s role in pancreatic cancer growth and metastasis via MAPK1-IQGAP1 phosphorylation

**Fig. 9:**
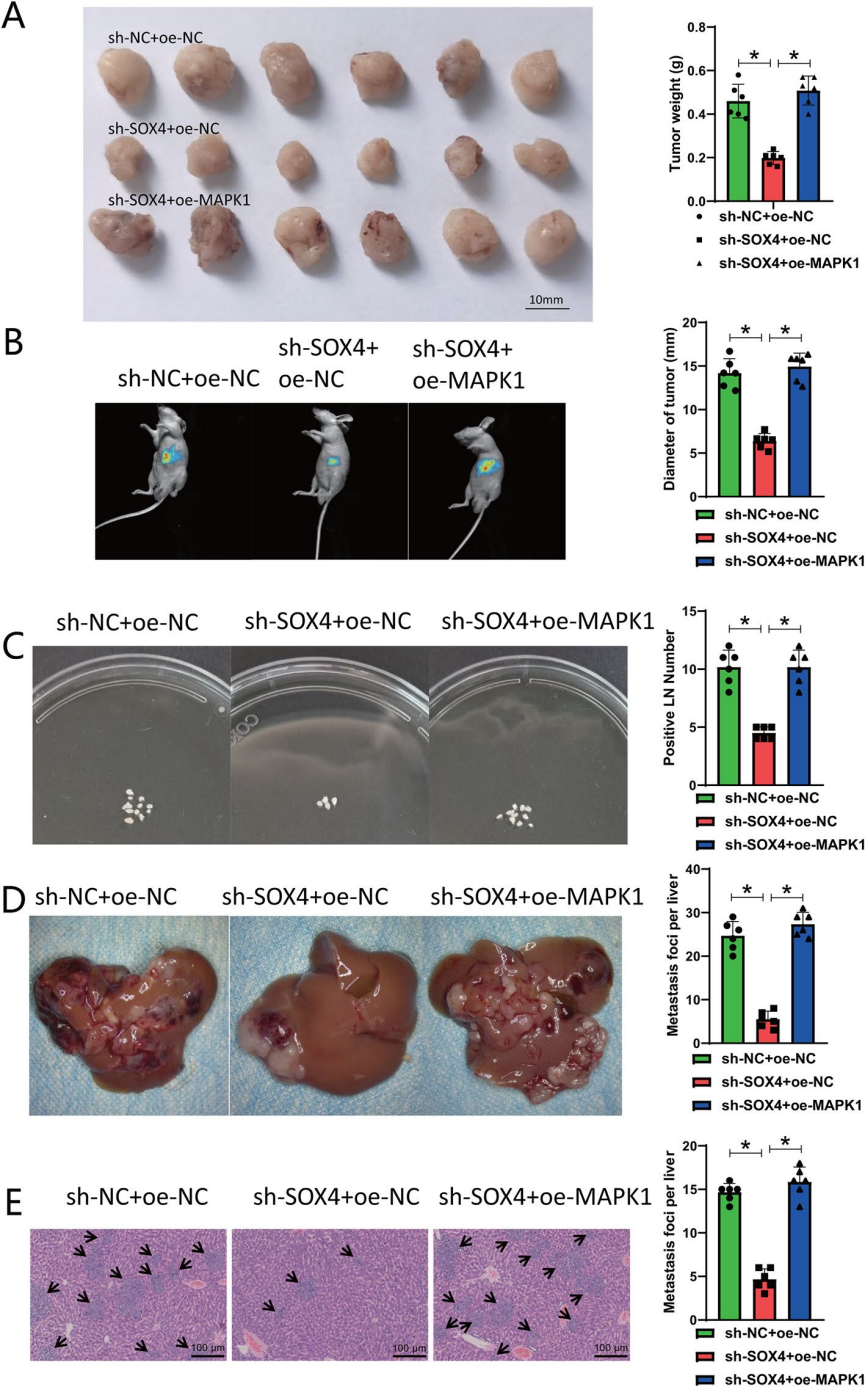
The influence of SOX4 on the growth and metastasis of pancreatic cancer through regulation of MAPK1-IQGAP1 phosphorylation. *Note *(**A**) Subcutaneous tumor volume (left) and weight (right) in nude mice of different groups; (**B**) In situ tumor images (left) and diameter analysis (right) in nude mice of different groups; (**C**) Statistical analysis of the number of axillary lymph node metastases in subcutaneous transplantation tumor in different groups, LN = Lymph node; (**D**) Statistical analysis of the average liver metastasis number in different groups of nude mice; (**E**) H&E staining to detect liver metastasis in different groups of nude mice, Scale bar = 1 mm, black arrows point to the liver metastasis region; * indicates a difference (*P* < 0.05), with six nude mice in each group

Lastly, we examined the impact of SOX4 on tumor proliferation and spread by establishing subcutaneous and orthotopic pancreatic cancer xenograft models, along with a liver metastasis model in nude mice in vivo. It was achieved by modulating the phosphorylation of MAPK1-IQGAP1. Upon silencing SOX4, we observed a suppression in the growth of subcutaneous and orthotopic tumors in nude mice. In contrast, the overexpression of MAPK1 enhanced the growth of subcutaneous and orthotopic tumors in nude mice (Fig. 9A-B). Further observations revealed that when SOX4 was silenced, there was a decrease in the number of metastases in axillary lymph nodes and the liver (Fig. 9C-E). Conversely, overexpressing MAPK1 led to an increase in the number of metastases in axillary lymph nodes and the liver. It has been observed that the inhibition of SOX4 could effectively suppress the growth and metastasis of pancreatic cancer by decreasing the phosphorylation level of MAPK1-IQGAP1.

## Discussion

**Fig. 10:**
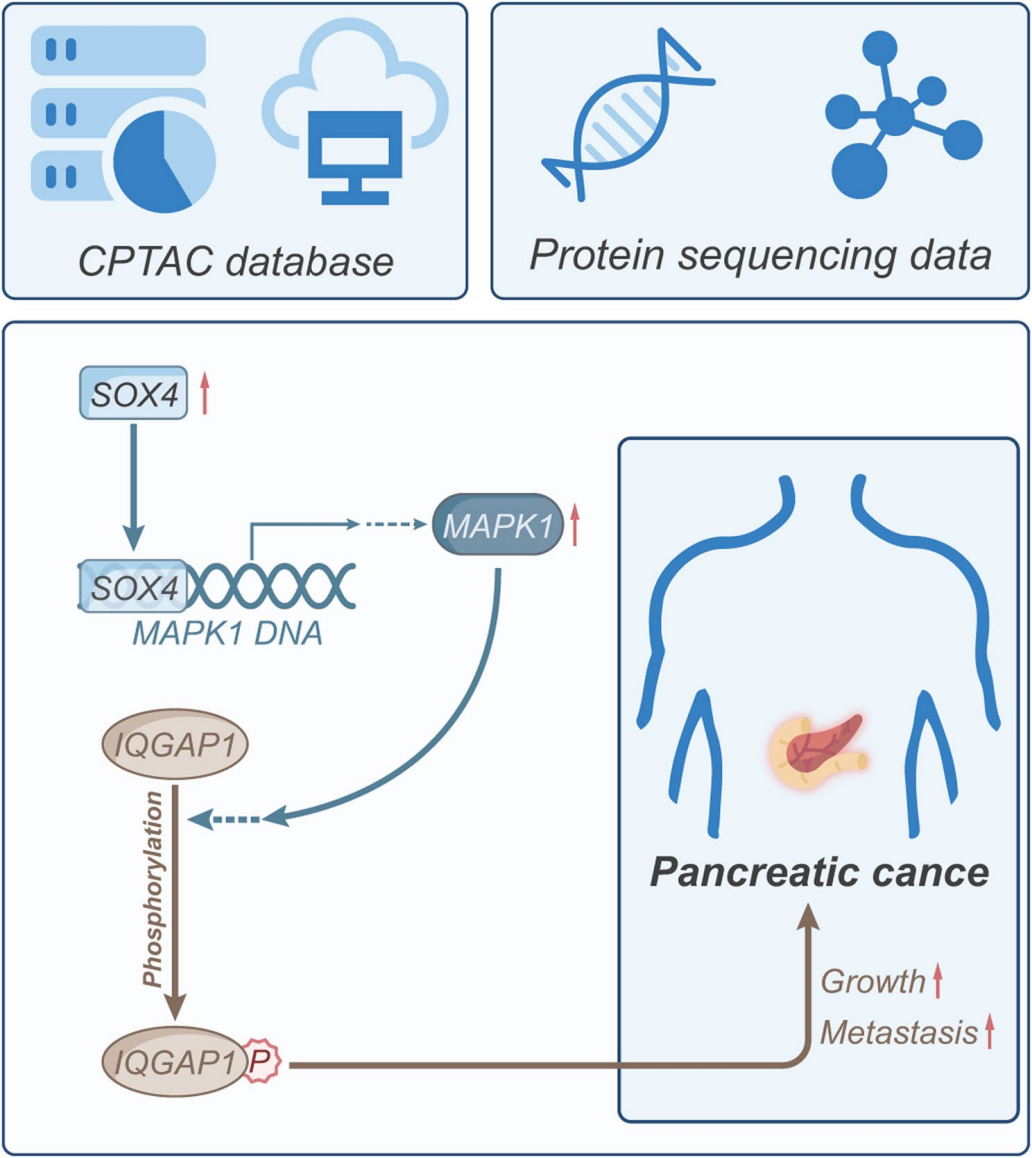
Schematic diagram illustrating the molecular mechanisms by which SOX4 regulates MAPK1-IQGAP1 phosphorylation to influence pancreatic cancer growth and metastasis

Based on the comprehensive research conducted above, we could tentatively deduce the following conclusion (Fig. 10): SOX4 can enhance the growth and metastasis of pancreatic cancer by activating the transcription of MAPK1, thereby facilitating the phosphorylation modification of IQGAP1. This study integrates the analysis of the CPTAC public database and tumor sequencing data from pancreatic cancer patients. Additionally, it verifies the findings through in vitro cell experiments and in vivo animal studies, thus yielding novel theoretical foundations and molecular targets for pancreatic cancer treatment.

This study revealed that SOX4 activates the transcription of MAPK1, thereby promoting the phosphorylation modification of IQGAP1, which is implicated in the growth and metastasis of pancreatic cancer. This finding is consistent with previous studies, suggesting that SOX4 functions as a promoter in various types of cancer [38–40]. Nevertheless, the specific role and regulatory mechanisms of {specific element} in pancreatic cancer remain inadequately documented. Our research provides new insights and evidence for this. Analysis of phosphoproteomics and proteomics data has revealed that MAPK1 could potentially play a pivotal role in the development of pancreatic cancer by promoting the phosphorylation modification of IQGAP1. This finding is in line with prior research, which has confirmed the significance of the MAPK signaling pathway as a critical regulator in various forms of cancer [41–43]. Nevertheless, the direct regulation of IQGAP1 by MAPK1 and its association with pancreatic cancer has not been thoroughly investigated. This study addresses this research gap.

The present study categorized patients with pancreatic cancer into two subtypes, C1 and C2, using proteomics data. Indeed, various cancer subtypes could yield varied impacts on treatment response and prognosis [44]. Specifically, our findings indicate that the high-risk C2 subtype is linked to a negative prognosis and a classification of high-grade malignant tumors. These findings provide valuable insights for developing future treatment strategies for pancreatic cancer. This study not only elucidated the regulatory effects of SOX4 on MAPK1 and IQGAP1 but also verified the underlying mechanisms through in vitro cell experiments and in vivo animal experiments. Simultaneously, the suppression of SOX4 hinders the proliferation, migration, and invasion of pancreatic cancer cells. This discovery establishes a theoretical foundation for considering SOX4 as a potential therapeutic target for treating pancreatic cancer.

While previous studies have implicated SOX4, MAPK1, and IQGAP1 in the occurrence and progression of cancer, reports are scarce on their specific relationship and interaction mechanisms. This study was the first to comprehensively and systematically explore these three factors’ roles and interaction mechanisms in pancreatic cancer. The findings provide new directions for pancreatic cancer research.

This study offers a comprehensive analysis of the role played by the phosphorylation regulatory axis of SOX4/MAPK1/IQGAP1 in the occurrence and development of pancreatic cancer. It provides novel insights into the molecular mechanisms underlying pancreatic cancer. Considering pancreatic cancer’s unfavorable prognosis and clinical treatment difficulties, this study could offer new targets for innovative therapeutic strategies. For instance, the regulatory axis involving SOX4 and MAPK1-IQGAP1 may offer guidance for treatment. Proteomics analysis categorizes pancreatic cancer patients into two subtypes, C1 and C2, laying the foundation for tailored treatment and prognosis assessment. The reliability of the results has been enhanced by confirming mechanistic findings through in vitro and in vivo experiments.

Despite obtaining a wealth of data from the CPTAC database, only samples from 8 patients with pancreatic cancer were utilized for experimental validation. This sample size may not be adequate to comprehensively represent the heterogeneity of pancreatic cancer patients. This study primarily utilized cell and animal models, and additional clinical trials are necessary to validate its practical application and effects in humans. The study stated that SOX4 promotes the phosphorylation of IQGAP1 by activating MAPK1 transcription. However, the precise phosphorylation mechanism and the regulatory factors require further investigation. In the future, it would be worth considering drug screening or treatment strategies that target the regulatory axis of SOX4 or MAPK1-IQGAP1. It could open up new possibilities for the treatment of pancreatic cancer. To enhance the sample size and obtain a more comprehensive understanding of the phosphorylation modification’s role in SOX4/MAPK1/IQGAP1 among diverse pancreatic cancer patients. Comprehensive mechanistic studies are necessary to examine the activation of MAPK1 transcription by SOX4 and the subsequent impact of MAPK1 on the phosphorylation of IQGAP1. Given the roles of SOX4, MAPK1, and IQGAP1 in other cancers, further investigation should be conducted to explore the functions and mechanisms of this regulatory axis in other cancer types.

## Conclusion

Overall, this study offers a fresh perspective on the mechanisms underlying pancreatic cancer and presents opportunities for exploring novel treatment approaches. However, additional research and experiments are still necessary to determine how these findings could effectively translate into clinical practice.

### Electronic supplementary material

Below is the link to the electronic supplementary material.

Supplementary Material 1

Supplementary Material 2

Supplementary Material 3

## Data Availability

The datasets generated and/or analyzed during the current study are available from the corresponding author on reasonable request.

## Abbreviations

LC-MS: Liquid chromatography-mass spectrometry
SPE: Solid-phase extraction
CPTAC: Clinical proteomic tumor analysis consortium
FDR: False discovery rate
KSEA: Kinase-substrate enrichment analysis
GSEA: Gene set enrichment analysis
GO: Gene ontology
KEGG: Kyoto encyclopedia of genes and genomes
WGCNA: Weighted gene co-expression network analysis
RT-qPCR: Real-time quantitative polymerase chain reaction
ChIP: Chromatin immunoprecipitation
PVDF: Polyvinylidene difluoride
HRP: Horseradish peroxidase
CCK-8: Cell counting kit-8
PBS: Phosphate-buffered saline
SPF: Specific pathogen free
H&E: Hematoxylin and eosin
SD: Standard deviation
ANOVA: Analysis of variance

## References

[1] Wu Z, Huang X, Cai M, Huang P, Guan Z (2022). Novel necroptosis-related gene signature for predicting the prognosis of pancreatic adenocarcinoma. Aging.

[2] Halbrook CJ, Lyssiotis CA, Pasca di Magliano M, Maitra A (2023). Pancreatic cancer: advances and challenges. Cell.

[3] Gan ZY, Callegari S, Cobbold SA et al. Activation mechanism of PINK1 [published correction appears in Nature. 2022;603(7903):E33]. Nature. 2022;602(7896):328–335. 10.1038/s41586-021-04340-2. 10.1038/s41586-021-04340-2 PMC8828467 34933320

[4] Kaiyrzhanov R, Mohammed SEM, Maroofian R (2022). Bi-allelic LETM1 variants perturb mitochondrial ion homeostasis leading to a clinical spectrum with predominant nervous system involvement. Am J Hum Genet.

[5] Sun M, Ge S, Li Z. The Role of Phosphorylation and Acylation in the Regulation of Drug Resistance in Mycobacterium tuberculosis. Biomedicines. 2022;10(10):2592. Published 2022 Oct 15. 10.3390/biomedicines10102592. 10.3390/biomedicines10102592 PMC9599588 36289854

[6] Ma Q, Zhang Q, Chen Y (2021). Post-translational modifications in oral Bacteria and their functional impact. Front Microbiol.

[7] Tian X, Wang R, Gu T et al. Costunolide is a dual inhibitor of MEK1 and AKT1/2 that overcomes osimertinib resistance in lung cancer. Mol Cancer. 2022;21(1):193. Published 2022 Oct 6. 10.1186/s12943-022-01662-1. 10.1186/s12943-022-01662-1 PMC9535870 36203195

[8] Mantini G, Pham TV, Piersma SR, Jimenez CR (2021). Computational Analysis of Phosphoproteomics Data in Multi-omics Cancer studies. Proteomics.

[9] Gerritsen JS, White FM (2021). Phosphoproteomics: a valuable tool for uncovering molecular signaling in cancer cells. Expert Rev Proteom.

[10] Qiao Z, Kong Y, Zhang Y (2022). Phosphoproteomics of extracellular vesicles integrated with multiomics analysis reveals novel kinase networks for lung cancer. Mol Carcinog.

[11] Sheykhi-Sabzehpoush M, Ghasemian M, Khojasteh Pour F (2023). Emerging roles of long non-coding RNA FTX in human disorders. Clin Transl Oncol.

[12] Osman MA, Sarkar FH, Rodriguez-Boulan E (2013). A molecular rheostat at the interface of cancer and diabetes. Biochim Biophys Acta.

[13] Liu J, Wang F, Luo F (2023). The role of JAK/STAT pathway in Fibrotic diseases: Molecular and Cellular mechanisms. Biomolecules.

[14] Kakodkar P, More S, András K (2020). Aspartic aminopeptidase is a novel biomarker of aggressive chronic lymphocytic leukemia. Cancers (Basel).

[15] Vikramdeo KS, Saha P, Dutta S (2020). Hyaluronan-binding protein 1 (HABP1) overexpression triggers induction of senescence in fibroblasts cells. Cell Biol Int.

[16] Liu W, Xie L, He YH et al. Large-scale and high-resolution mass spectrometry-based proteomics profiling defines molecular subtypes of esophageal cancer for therapeutic targeting. Nat Commun. 2021;12(1):4961. Published 2021 Aug 16. 10.1038/s41467-021-25202-5. 10.1038/s41467-021-25202-5 PMC8368010 34400640

[17] Satpathy S, Krug K, Jean Beltran PM (2021). A proteogenomic portrait of lung squamous cell carcinoma. Cell.

[18] Jiang Z, Zhang W, Zeng Z (2022). A comprehensive investigation discovered the novel methyltransferase METTL24 as one presumably prognostic gene for kidney renal clear cell carcinoma potentially modulating tumor immune microenvironment. Front Immunol.

[19] Zhang J, Pan S, Han C (2022). Combination of Immune-Related Network and Molecular Typing Analysis defines a three-gene signature for Predicting Prognosis of Triple-negative breast Cancer. Biomolecules.

[20] Wu ZX, Huang X, Cai MJ, Huang PD, Guan Z (2022). Development and validation of a Prognostic Index based on genes participating in Autophagy in patients with lung adenocarcinoma. Front Oncol.

[21] Yuan F, Lu L, Zhang Y, Wang S, Cai YD (2018). Data mining of the cancer-related lncRNAs GO terms and KEGG pathways by using mRMR method. Math Biosci.

[22] Jiang BC, He LN, Wu XB (2017). Promoted Interaction of C/EBPα with demethylated Cxcr3 gene promoter contributes to Neuropathic Pain in mice. J Neurosci.

[23] Wang M, Liu J, Zhao Y et al. Upregulation of METTL14 mediates the elevation of PERP mRNA N^6^ adenosine methylation promoting the growth and metastasis of pancreatic cancer. Mol Cancer. 2020;19(1):130. Published 2020 Aug 25. 10.1186/s12943-020-01249-8. 10.1186/s12943-020-01249-8 PMC7446161 32843065

[24] Sun J, Shen H, Shao L (2020). HIF-1α overexpression in mesenchymal stem cell-derived exosomes mediates cardioprotection in myocardial infarction by enhanced angiogenesis. Stem Cell Res Ther.

[25] Taniue K, Kurimoto A, Takeda Y (2016). ASBEL-TCF3 complex is required for the tumorigenicity of colorectal cancer cells. Proc Natl Acad Sci U S A.

[26] Nelson JD, Denisenko O, Sova P, Bomsztyk K. Fast chromatin immunoprecipitation assay. Nucleic Acids Res. 2006;34(1):e2. Published 2006 Jan 5. 10.1093/nar/gnj004. 10.1093/nar/gnj004 PMC1325209 16397291

[27] Liu L, Xie D, Xie H (2019). ARHGAP10 inhibits the proliferation and metastasis of CRC cells via blocking the activity of RhoA/AKT signaling pathway. Onco Targets Ther.

[28] Huang YF, Niu WB, Hu R et al. FIBP knockdown attenuates growth and enhances chemotherapy in colorectal cancer via regulating GSK3β-related pathways [published correction appears in Oncogenesis. 2022;11(1):36]. Oncogenesis. 2018;7(9):77. Published 2018 Oct 2. 10.1038/s41389-018-0088-9. 10.1038/s41389-018-0088-9 PMC6167373 30275459

[29] Hou Y, Zhang Q, Pang W (2021). YTHDC1-mediated augmentation of miR-30d in repressing pancreatic tumorigenesis via attenuation of RUNX1-induced transcriptional activation of Warburg effect. Cell Death Differ.

[30] Han F, Liu WB, Shi XY (2018). SOX30 inhibits Tumor Metastasis through attenuating wnt-signaling via Transcriptional and Posttranslational Regulation of β-Catenin in Lung Cancer. EBioMedicine.

[31] Needham EJ, Hingst JR, Parker BL (2022). Personalized phosphoproteomics identifies functional signaling. Nat Biotechnol.

[32] Li C, Sun YD, Yu GY (2020). Integrated Omics of Metastatic Colorectal Cancer. Cancer Cell.

[33] Smith JM, Hedman AC, Sacks DB (2015). IQGAPs choreograph cellular signaling from the membrane to the nucleus. Trends Cell Biol.

[34] Peng X, Wang T, Gao H (2021). The interplay between IQGAP1 and small GTPases in cancer metastasis. Biomed Pharmacother.

[35] Meng D, Wu W, Li Z, Qin G (2015). IQGAP1 modulates the proliferation and invasion of thyroid cancer cells in response to estrogen. Int J Mol Med.

[36] Wang H, Huo X, Yang XR (2017). STAT3-mediated upregulation of lncRNA HOXD-AS1 as a ceRNA facilitates liver cancer metastasis by regulating SOX4. Mol Cancer.

[37] Wang N, Liu W, Zheng Y (2018). CXCL1 derived from tumor-associated macrophages promotes breast cancer metastasis via activating NF-κB/SOX4 signaling. Cell Death Dis.

[38] Guo M, Lin B, Li G, Lin J, Jiang X (2020). LncRNA TDRG1 promotes the proliferation, migration, and invasion of cervical cancer cells by sponging mir-214-5p to target SOX4. J Recept Signal Transduct Res.

[39] Hanieh H, Ahmed EA, Vishnubalaji R, Alajez NM (2020). SOX4: epigenetic regulation and role in tumorigenesis. Semin Cancer Biol.

[40] Moreno CS (2020). SOX4: the unappreciated oncogene. Semin Cancer Biol.

[41] Luo J (2021). KRAS mutation in pancreatic cancer. Semin Oncol.

[42] Lee S, Rauch J, Kolch W (2020). Targeting MAPK signaling in Cancer: mechanisms of Drug Resistance and Sensitivity. Int J Mol Sci.

[43] Guo YJ, Pan WW, Liu SB, Shen ZF, Xu Y, Hu LL (2020). ERK/MAPK signalling pathway and tumorigenesis. Exp Ther Med.

[44] Chakravarty D, Solit DB (2021). Clinical cancer genomic profiling. Nat Rev Genet.

