# Supplementary figures and images for: Deciphering the SOX4/MAPK1 regulatory axis: a phosphoproteomic insight into IQGAP1 phosphorylation and pancreatic Cancer progression
Source: J Transl Med. 2024 Jun 28;22:602. doi: 10.1186/s12967-024-05377-3 (PMC11212360; doi:10.1186/s12967-024-05377-3)

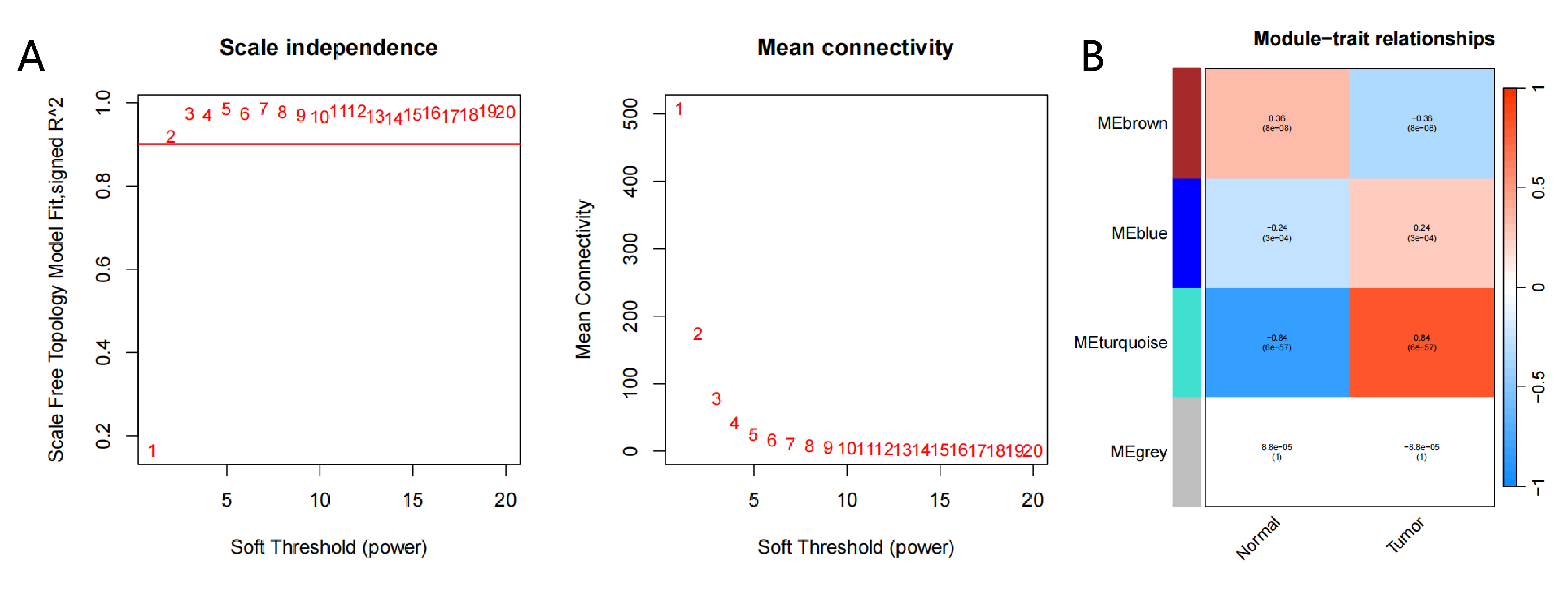

Supplement: Supplementary file 1 — Supplementary Material 1 [file 12967_2024_5377_MOESM1_ESM.jpg]

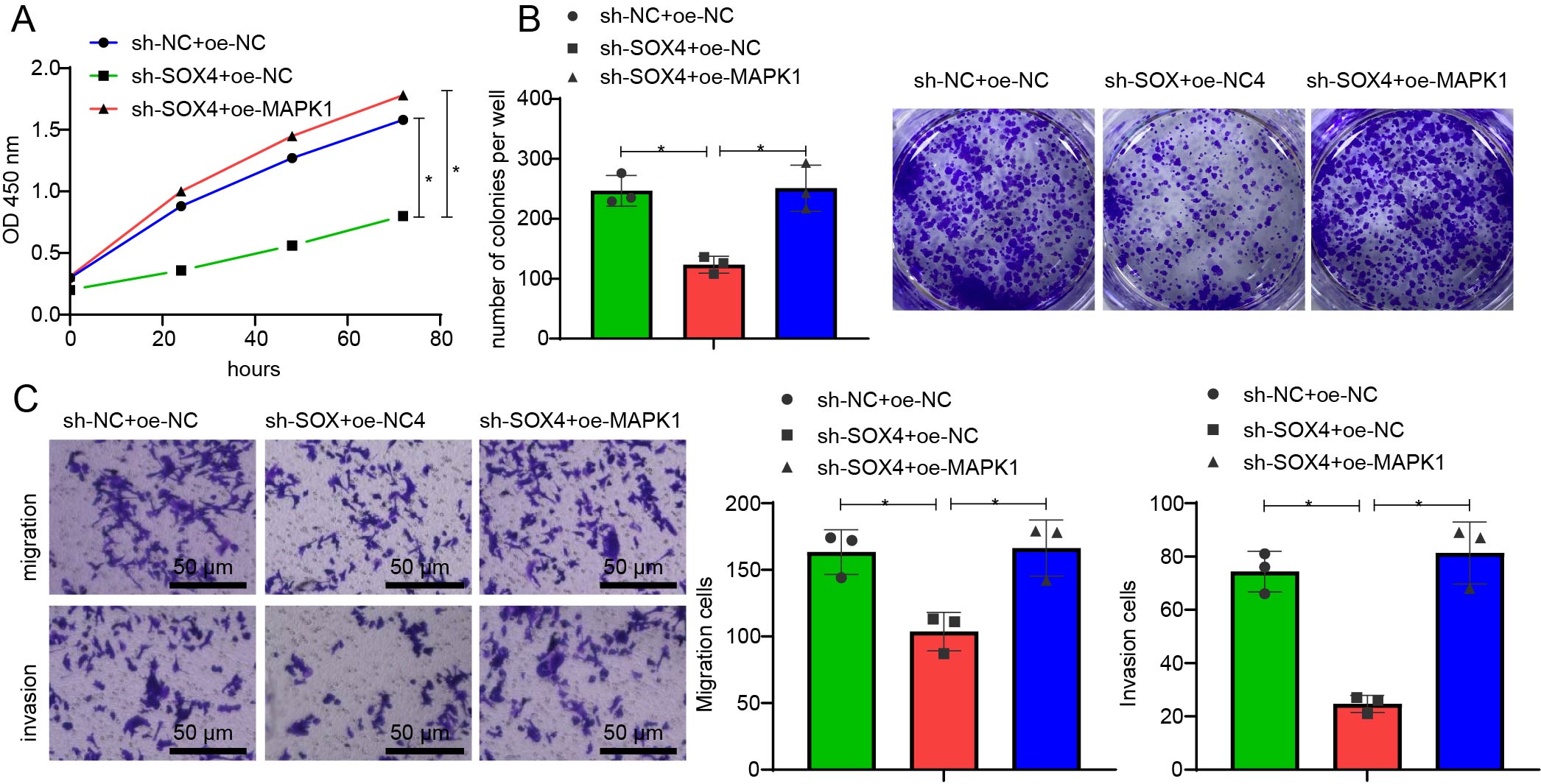

Supplement: Supplementary file 2 — Supplementary Material 2 [file 12967_2024_5377_MOESM2_ESM.jpg]
